# ER–Mitochondria Microdomains in Cardiac Ischemia–Reperfusion Injury: A Fresh Perspective

**DOI:** 10.3389/fphys.2018.00755

**Published:** 2018-06-15

**Authors:** Hao Zhou, Shuyi Wang, Shunying Hu, Yundai Chen, Jun Ren

**Affiliations:** ^1^Chinese People’s Liberation Army General Hospital, People’s Liberation Army Medical School, Beijing, China; ^2^Center for Cardiovascular Research and Alternative Medicine, University of Wyoming College of Health Sciences, Laramie, WY, United States; ^3^Department of Cardiology, Zhong Shan Hospital, Fudan University, Shanghai, China

**Keywords:** ER–mitochondria microdomains, ischemia/reperfusion injury, mitochondrial fission, mitophagy, oxidative stress, calcium signaling, cell death

## Abstract

The mitochondrial and endoplasmic reticulum (ER) homeostasis is pivotal to the maintenance of an array of physiological processes. The physical contact and association between ER and mitochondria, known as the ER–mitochondria microdomains or mitochondria-associated ER membrane (MAM), temporally and spatially regulates the mitochondria/ER structure and function. More evidence suggests a role for MAMs in energy production, cellular contraction and mobility, and normal extracellular signal transmission. In pathological states, such as cardiac ischemia–reperfusion (I/R injury), this ER–mitochondria microdomains may act to participate in the cellular redox imbalance, ER stress, mitochondrial injury, energy deletion, and programmed cell death. From a therapeutic perspective, a better understanding of the cellular and molecular mechanisms of the pathogenic ER–mitochondria contact should help to identify potential therapeutic target for cardiac I/R injury and other cardiovascular diseases and also pave the road to new treatment modalities pertinent for the treatment of reperfusion damage in clinical practice. This review will mainly focus on the possible signaling pathways involved in the regulation of the ER–mitochondria contact. In particular, we will summarize the downstream signaling modalities influenced by ER–mitochondria microdomains, for example, mitochondrial fission, mitophagy, calcium balance, oxidative stress, and programmed cell death in details.

## Introduction

Myocardial infarction (MI) is one of the leading causes of mortality worldwide due to acute occlusion of coronary arteries. Although revascularization treatment has offered proven protective efficacy for patients with MI, it also yields undesired ischemia–reperfusion (I/R) injury following the restoration of epicardial blood flow (Nunez-Gomez et al., 2017; Zhou et al., 2018b). A number of scenarios have been postulated for I/R injury, including oxidative stress, calcium imbalance, mitochondrial damage, excessive inflammation response, endoplasmic reticulum (ER) stress, and programmed cell death (Du et al., 2017; Garcia-Nino et al., 2017; Harisseh et al., 2017; Jahandiez et al., 2017). These culprit factors unfortunately lead to a secondary damage to the heart and thus compromise the clinical benefits from revascularization therapy (Merjaneh et al., 2017; Rienks et al., 2017). Notably, mitochondrial damage and ER stress have been well recognized as major upstream factors governing the progression of cardiac I/R injury. Thereby, the structural and functional association between mitochondria and ER has emerged as an area of intensive research that has evolved rapidly over the last decade (Pihan et al., 2017).

**FIGURE 1:**
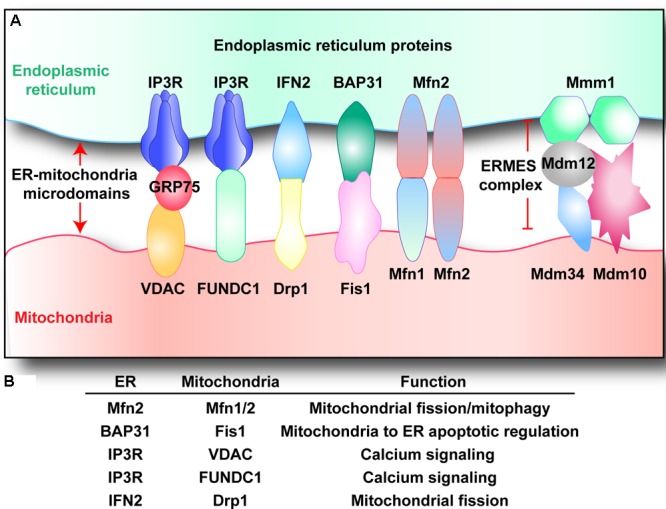
**(A,B)** Endoplasmic reticulum–mitochondria microdomains complexes. Multiple structures that tether mitochondria with ER have been described. Voltage-dependent anion channel (VDAC) and inositol 1,4,5-trisphosphate receptor (IP3R) interacts via GRP75, regulating calcium balance between mitochondria and ER. Similarly, IP3R also cooperates with FUN14 domain-containing protein 1 (FUNDC1), modifying mitochondrial calcium homeostasis. B-cell receptor associated protein 31 (BAP31) binds to mitochondrial fission 1 protein (Fis1), regulating cellular apoptosis. Inverted formin-2 (INF2) interacts with dynamin-related protein 1 (Drp1), handling mitochondrial fission. ER-located mitofusin 2 (Mfn2) interacts with mitochondrial Mfn1/Mfn2, controlling mitochondrial fission and mitophagy. Besides, the ER–mitochondria encounter structure (ERMES) complex is composed of: the OMM proteins Mdm10 and Mdm34, the ER protein Mmm1, and the cytosolic protein Mdm12.

The existence of physical links between ER and mitochondria have been suggested based on co-sedimentation of ER particles with mitochondria and electron microscopic observations of close associations between mitochondria and ER vesicles (Shore and Tata, 1977; Meier et al., 1981; Mannella et al., 1998). ER–mitochondrial microdomains [termed as the mitochondria-associated membranes (MAMs)] are purportedly comprised of a variety of proteins including, but not limited to, (i) the inositol 1,4,5-trisphosphate receptors (IP3R) on the ER and voltage-dependent anion-selective channel protein (VDAC) located on the mitochondria, through GRP75, which play a role in calcium signaling; (ii) the mitofusin 2 (Mfn2) located in the ER and other molecular chaperones such as mitofusin 1 (Mfn1) and FUNDC1 in the mitochondria, that play a role in tethering and modulating mitochondrial dynamics; (iii) the ER stress sensor PERK that initiates signaling in response to ER stress; and (iv) many more others including ryanodine receptor Ca^2+^ channel (RyR) (Chen et al., 2012), AMF-R (Wang et al., 2000), Miro1 (Fransson et al., 2003), BAP31 (Iwasawa et al., 2011), Fis1 (Wang et al., 2011; **Figures 1A,B**).

Structural and functional interactions of mitochondria with the ER have been demonstrated for rat hearts (Ruiz-Meana et al., 2010; Fernandez-Sanz et al., 2014; Gomez et al., 2016) and the distance between the ER and the outer mitochondrial membrane (OMM) is originally estimated to be approximately 100 nm (Soltys and Gupta, 1992; Mannella et al., 1994). However, a more recent study using electron tomography demonstrated that the minimum distance is much less, 10 nm at the smooth ER and 25 nm at the rough ER (Csordas et al., 2006). The physical cooperation between the ER and mitochondria offers pivotal roles in several aspects of cellular functions, including Ca^2+^ signaling, lipid transport, energy metabolism, and cellular survival (Honrath et al., 2018). However, in response to stress response, especially cardiac I/R injury, ER–mitochondria contact converts mitochondria and ER from ATP providers and protein factories that energize the cell to agents of cell death, respectively. Here, this mini-review is intended to summarize the current contemporary understanding with regards to the casual role of ER–mitochondrial microdomains in the onset and development of myocardial I/R injury.

## Mitochondrial Fission

Although commonly depicted as shuttle-shaped structures, mitochondria form a highly dynamic network within cardiomyocyte where they constantly undergo the fission and fusion processes (Lopez-Crisosto et al., 2017; Wang et al., 2017). The mitochondrial fission could be apparently noted in cardiac I/R injury (Gao et al., 2016; Cowan et al., 2017; Maneechote et al., 2017; Nan et al., 2017; Zhou et al., 2018e), and the aim of mitochondrial fission is to generate more daughter mitochondria that meet the cardiomyocyte demand in ischemic stage and/or in reperfusion phase. Under physiological conditions, moderate mitochondrial fission allows the dissemination of various metabolites and macromolecules throughout the entire compartment (Westermann, 2012). At the same time, mitochondrial fission is required for the removal of damaged and inactive organelles by way of autophagy (Twig et al., 2008a,b). When the bioenergetic state becomes critical, for example under nutrient deprivation (Sauvanet et al., 2010; Toyama et al., 2016), exercise (Coronado et al., 2018), or exposure to certain forms of stress (Theurey and Rieusset, 2017), fission is turned on to optimize mitochondrial function. However, excessive mitochondrial fission has been suggested as a primary causative factor in the pathogenesis of myocardial reperfusion injury based on succinct studies from independent laboratories (Ong et al., 2010; Sharp et al., 2014) including ourselves (Zhou et al., 2017a, 2018c,f; Jin et al., 2018). At the molecular levels, mitochondrial fission is exclusively governed by dynamin-related 1 (Drp1) and its adaptors such as mitochondrial fission factor (Mff) and mitochondrial fission 1 protein (Fis1) which help Drp1 tightly dock on mitochondria and then assist Drp1 to form the contractile ring around mitochondria (Garcia-Nino et al., 2017; Hong et al., 2017; Zhou et al., 2017c). Interestingly, recent research has depicted that ER–mitochondria microdomain closely wraps around the mitochondria and initiates a mitochondrial constriction at the contact sites before Drp1 is recruited to trigger mitochondrial fission (Friedman et al., 2011). Besides, Drp1 is also found to assemble on mitochondria preferentially at sites of the ER–mitochondria contact (Westermann, 2011), suggesting that ER–mitochondria microdomain may play an active role in the early stages of mitochondrial fission via defining the division sites. Thereby, these effects may aggravate the cardiac I/R injury through Drp1 recruitment and constriction. Besides, earlier work by Korobova et al. (2013) also observed a similar action for the ER–mitochondria microdomain on mitochondrial division, suggesting that repression of ER–mitochondria communication may provide more benefits for cardiac I/R injury via disrupting mitochondrial fission. Notably, Korobova et al. (2013) further pointed out that the ER-bound protein inverted formin 2 (INF2) predominantly controls mitochondrial fission possibly by forming a constrictions ring prior to translocation of Drp1 onto the mitochondrial membrane. More importantly, INF2 interacts with the calcium-binding protein calmodulin, which empowers the ER–mitochondria microdomain to shape the local calcium homeostasis (Wales et al., 2016). This regulatory mechanism amplifies the intracellular calcium delivery from ER to mitochondria, ensuring the success of mitochondrial fission, which would need sufficient Ca^2+^ to complete the organelle contraction. Following studies further confirm that INF2 also enhances actin polymerization on the ER (Gurel et al., 2015; Chakrabarti et al., 2018), which facilitates mitochondrial division through actin-dependent mitochondrial contractile.

Interestingly, the actin polymerization mediated by INF2 could in turn increases the ER–mitochondria contact area, as assessed by electron microscopy (Steffen and Koehler, 2018). These observations propose a positive feedback between ER–mitochondria microdomain and mitochondrial fission; ER–mitochondria microdomain first establishes the potential contractile site for mitochondria fission via INF2, which in turn further narrows the distance between ER and mitochondria, leading to a progressive amplification of fission signals. Based on this, we question whether the distance between ER and mitochondria may serve as an early hallmark for the extent of mitochondrial fission and cardiac I/R injury. More work is needed to verify this hypothesis. Besides, another study notes a reduction in mitochondrial diameter at sites in which the ER is almost completely wrapped around the mitochondrial membrane (from ∼210 nm for uncircumscribed mitochondria to ∼140 nm for circumscribed mitochondria) (Friedman et al., 2011; Kang et al., 2017). In other words, the area of ER–mitochondria microdomain is positively correlated with the extent mitochondrial fission.

Besides, other components of ER–mitochondria microdomain are also reported to engage in mitochondrial division. Knockdown of mitochondrial calcium uniporter (MCU) interrupted mitochondrial fission, and Chakrabarti et al. (2018) noted a 2.5-fold decrease in the fission event in cells lacking MCU. Subsequent studies have identified the casual relationship between MCU activation and cardiac I/R injury. Using myocardial reperfusion model, inhibition of MCU via genetic ablation or pharmacological inhibition sustains myocardial contractile function (Kwong et al., 2015), alleviates necroptosis and apoptosis levels by 30 and 50%, respectively (Oropeza-Almazan et al., 2017). In addition, inhibition of MCU represses caspase-3/-7/-8/-9 activation (Oropeza-Almazan et al., 2017), interrupts calcium imbalance (Seidlmayer et al., 2015), maintains mitochondria oxygen consumption rates (Rasmussen et al., 2015), preserves mitochondrial potential (Rasmussen et al., 2015), reduces cellular ROS generation (Rasmussen et al., 2015), and blocks the opening of mitochondrial permeability transition pore (mPTP) (Luongo et al., 2015). These findings have highlighted that the activity of MCU in ER–mitochondria microdomain is highly responsible for mitochondrial anomalies and cardiomyocyte injury induced by I/R injury, and support the concept of MCU inhibition as a potential therapeutic strategy.

Besides, mitochondrial fission may also be regulated by mitofusins expressed within the ER–mitochondria microdomain. Structurally, Mfn1 and Mfn2 both localize predominantly on the OMM, whereas the latter also expresses on ER and ER–mitochondria microdomain (Koshiba et al., 2004). Genetic ablation of Mfn1 or Mfn2 results in embryonic lethality, suggesting essential developmental roles for both isoforms (Chen et al., 2003, 2007). Mechanistically, the expression of ER-located Mfn2 is crucial for tethering the ER to the mitochondria and thus stabilizing ER–mitochondria microdomain formation via tight interaction with mitochondrial Mfn1 and forming the Mfn1–Mfn2 heteromultimer (de Brito and Scorrano, 2008). Recent studies have found that Mfn2 deletion attenuates cardiac cell death in response to I/R injury and the potential to undergo calcium-dependent mitochondrial permeability transition (Papanicolaou et al., 2011). This observation is further confirmed by a report that adult murine heart deficient in both Mfn1 and Mfn2 is protected against acute cardiac I/R injury (Hall et al., 2016), a finding which is associated with defects in mitochondrial fission and reduced mitochondrial calcium overload, suggesting that mitofusins, regulated by ER–mitochondria microdomain, is of importance to promote the progression of cardiac I/R injury. Taken together, owing to the direct contact of ER and mitochondria, the ER–mitochondria microdomain are easy to cope with mitochondrial fission via pleiotropic molecular mechanisms on the one hand, and that they have ideally “guard” roles to prevent cardiac I/R injury on the other hand. Accordingly, it seems likely that inhibition of the key site in ER–mitochondria microdomain may prove as an effective pharmacological intervention for reducing the severity of cardiac I/R damage via interrupting lethal mitochondrial fission.

## Mitophagy

Mitophagy, a kind of mitochondrial autophagy, sweeps the damaged mitochondria and provides the nutrients necessary to preserve cell viability via timely removal of poor-structured mitochondria with the assistance of lysosome (Goiran et al., 2018; Lindqvist et al., 2018). Recent reports suggested that autophagosome membrane may be derived primarily from the ER (Molino et al., 2017; Song et al., 2018). This notion is also confirmed by an observation that pre-autophagosome/autophagosome marker ATG14 re-localizes to the ER–mitochondria contact site after starvation (Hamasaki et al., 2013). This means ATG14, an indispensable factor for mitophagy activation, is actually regulated by ER–mitochondria microdomain. Interestingly, in the cardiac myocardial infraction or coronary artery disease model, ATG14 is required for the angiogenesis via Becn1–Vps34–ATG14 complex (Lu et al., 2016), which is a novel agent for treatment of acute ischemia-mediated myocardial injury via handling revascularization. This observation is further verified by Liu et al. (2017) using cardiomyocyte hypoxia-reoxygenation (HR) model. These investigators found that ATG14 unfortunately decreases in response to HR stimulus as a result of elevated microRNA-130a, and reintroduction of ATG14 via inhibition of microRNA-130a attenuates HR-mediated cardiomyocyte apoptosis. Taken together, these pieces of evidence have pointed out that ATG14 cooperates with ER–mitochondria microdomain to ensure mitophagy activation which provides pro-survival signals for the damaged hearts.

Besides, other studies further revealed that the pro-autophagic proteins BECN1/Beclin1 are both found to re-localize at ER–mitochondria microdomain, where they enhance the ER–mitochondria interaction along with increased mitophagy activity (Gelmetti et al., 2017). Ample evidence has depicted a protective function of Beclin1 on cardiac I/R injury. In particular, Beclin1 expression is downregulated after reperfusion injury (Dai et al., 2017), although it is significantly upregulated by ischemia preconditioning (Xie et al., 2018). Restoration of Beclin1 attenuates HR-mediated cardiomyocyte death (Ma et al., 2012). At the molecular levels, Beclin1 activation is involved in stress protein degradation (Fuhrmann and Brune, 2017), ROS clearance (Sun et al., 2017), inflammation repression (Chi et al., 2018), and eNOS-dependent vasodilation restoration (Zhang J.X. et al., 2018). Overall, the above information comprehensively validated the permissive role for Beclin1 in cardiac I/R injury, which acts as an upstream mediator for mitophagy via cooperation with ER–mitochondria microdomain.

Notably, a recent study from Gautier et al. (2016) has provided some new insights into the interactive mechanism for mitophagy and ER–mitochondria microdomain. In patients with Parkinson’s disease or Parkin-knockout mice, ER and mitochondria seem to be in closer proximity, followed by excessive calcium flux to the cytosol partly owing to the enhanced ER-to-mitochondria Ca^2+^ transfers. This finding has indicated that loss of mitophagy receptor fosters ER to move too close to the mitochondria, which unexpectedly contributes to the calcium leakage into cytoplasm and subsequent neurodegeneration. These data highlight that mitophagy is highly manipulated by ER–mitochondria microdomain on the one hand, and it also in turn corrects the excessive ER–mitochondria contact in a Parkin-dependent manner which could be considered as a negative feedback response to ensure the moderate ER–mitochondria communication. However, the negative feedback reaction has not been identified in cardiac I/R injury and thus more works are required to provide several evidences for this.

In spite of the extensive research which has been carried out over the past decades to figure out the molecular feature of mitophagy in cardiac I/R injury, the precise action of mitophagy in acute cardiomyocyte damage still remains elusive (Zhou et al., 2018b). Interestingly, the upstream regulatory mechanism for mitophagy is well-documented. There are three adaptors identified as the mitophagy inducer including Parkin, BCL2/adenovirus E1B 19 kDa protein-interacting protein 3 (Bnip3), and FUN14 domain containing 1 (FUNDC1). Interestingly, those adaptors could signal distinct mitophagic response for cardiomyocytes fate in I/R injury ranging from survival to death based on recent studies. Briefly, Binp3-mediated mitophagy is harmful for reperfused heart through turning on mitochondrial death (Jin et al., 2018). Similarly, the Parkin-dependent mitophagy also promoted mitophagy activity which unfortunately consumes most mitochondria, leading to the energy depletion and cell death (Zhou et al., 2017d). Interestingly, FUNDC1-related mitophagy is primarily activated by ischemic preconditioning and confers the protection against reperfusion injury (Zhou et al., 2017b,e, 2018f). Our finding is also supported by several in-depth studies in different disease models such as fatty liver disease and cancer (Chen et al., 2017; Li et al., 2018; Shi et al., 2018; Zhou et al., 2018a).

Recently, a delicate work from Wu et al. (2017) demonstrated that FUNDC1 could bind to IP3Rs to form the ER–mitochondria microdomain, which modulates ER–mitochondria Ca^2+^ exchange, mitochondrial fission, and mitophagy. Genetic ablation of FUNDC1 downregulates the levels of IP3R, disrupts ER–mitochondria microdomain contact, and worsens cardiac function in cardiac I/R model. This work has identified, for the first time, the FUNDC1, a mitophagy-related protein, as an integral component of ER–mitochondria microdomain, redefining the paradigm between ER–mitochondria microdomain and mitophagy regulation. Notably, mitofusins, the indispensable elements for ER–mitochondria microdomain as our mentioned above, have recently been suggested in the recycling of mitochondria content during starvation-induced autophagy (Marchi et al., 2014; Tubbs and Rieusset, 2017). Disruption of ER–mitochondria microdomain by Mfn2 deletion inhibits mitophagy and thus increases the vulnerability of heart to I/R challenge (Zhao et al., 2012) due to extensive accumulation of autophagosomes. This observation is also subsequently supported by other studies that Mfn2 is required for protective mitophagy activation and cardioprotection in the setting of I/R injury (Campos et al., 2016). At the molecular levels, Mfn2 tethers the mitochondrial outer membrane to the ER, and this effect facilitates the transfer of phosphatidylserine from the ER to mitochondria, which in turn, is required for phosphatidylethanolamine production employed in autophagosome membrane formation (Hailey et al., 2010). Notably, these data indicate that ER–mitochondria microdomain-located Mfn2 has the ability to activate protective mitophagy which sends the pro-survival signals for reperfused heart. Accordingly, several researchers suggest that activation of Mfn2-dependent mitophagy would provide more benefits for cardiomyocyte under I/R stress (Zhang W. et al., 2018).

However, this conclusion seems to oppose to the observations that Mfn2 deletion attenuates cardiac cell death in response to I/R injury via modifying mitochondrial fission, as we summarized above. To explain the plausibly inconsistent results, several key points need to be emphasized. One is that the fatal fission activated by ER–mitochondria microdomain is heavily relying on the formation of Mfn1–Mfn2 heteromultimer; the former expressed on ER and the latter located on mitochondria. However, the protective mitophagy modified by ER–mitochondria microdomain is only dependent on Mfn2 rather than the Mfn1–Mfn2 heteromultimer. Considering that pro-apoptotic fission is excessively activated, whereas pro-survival mitophagy is mostly inhibited at the stage of I/R injury, we ask whether increased mitochondrial fission “over-consumes” Mfn2 via establishing links between Mfn1 and Mfn2 heteromultimer, leading to the failure of deficient Mfn2 to trigger mitophagy. Last but not the least, the functional role of Mfn2 in mitophagy activation is to help the phospholipid transfer from ER to mitochondria, promoting the formation of autophagosome membrane. However, due to the Mfn1–Mfn2 interaction, decreased Mfn2 monomer in ER–mitochondria microdomain is by no means capable of initiating mitophagy. Collectively, although ER–mitochondria microdomain-located Mfn2 could activate the protective mitophagy to enhance the heart resistance to I/R injury, it is unfortunately employed by mitochondrial fission, leading to the increased mitochondrial fission and decreased mitophagy. This information may lay the foundation to help us understand the paradoxical role of Mfn2 in cardiac I/R injury. Nonetheless, further work to illustrate the potential pleiotropic effects of Mfn2 on I/R injury via balancing fission and mitophagy are required to obtain more comprehensive picture of ER–mitochondria microdomain in cardiomyocyte fate under acute reperfusion stress.

## Cellular Calcium Balance

The enzymes involved in the tricarboxylic acid (TCA) cycle and the mitochondrial respiratory complex are critically dependent on the moderate rise in mitochondrial Ca^2+^ levels to maintain cellular bioenergetics and meet the cell demand via ATP generation (Boone et al., 2017; Fuhrmann and Brune, 2017; Torres-Estay et al., 2017). Subsequently, with the assistance of mitochondria-produced ATP, ER rapidly releases Ca^2+^ into cytoplasm where Ca^2+^ interacts with troponin and ensures the cardiomyocyte beating and myocardial contraction (Eisner et al., 2017; Merjaneh et al., 2017; Mughal et al., 2018). Notably, excessive mitochondrial Ca^2+^ uptake leads to mitochondrial dysfunction and initiation of a cascade of pro-apoptotic events. The checkpoint for this phenomena lies on the ER–mitochondria microdomain (Dreser et al., 2017). The calcium handling proteins, RyRs (excitable cells) and IP3Rs (non-excitable cells) on the ER, as well as VDAC and MCU on the outer and inner mitochondrial membranes (Ligeza et al., 2017), respectively, have been shown to reside in close proximity at this interface of ER–mitochondria microdomain where they function to help the facile transfer of Ca^2+^ from the ER to mitochondria. Mechanistically, a high microdomain Ca^2+^ levels may be shaped after IP3Rs opening and the microdomain Ca^2+^ is largely buffered by mitochondria via MCU. Besides, a recent study also demonstrates that VDAC1 is structurally and physically linked to the type-1 IP3R through the molecular chaperone Grp75 (Szabadkai et al., 2006) and facilitates the Ca^2+^ communication between mitochondria and ER. Notably, those two Ca^2+^-exchange mechanisms regulated by ER–mitochondria microdomain are also noted in cardiac I/R injury. First, it is generally believed that Ca^2+^ should flow easily through VDAC channels because VDAC shows only a weak selectivity for small monovalent ions (Colombini, 1980; Hodge and Colombini, 1997). Per recent findings, acute myocardial reperfusion injury promotes VDAC phosphorylation (Schwertz et al., 2007) and this process is mainly regulated by glycogen synthase kinase (GSK)-3 or Akt (Das et al., 2008). Inhibition of VDAC phosphorylation by GSK-3 inhibitors is beneficial for reperfused heats (Das et al., 2008). At the molecular levels, two mechanisms involved in this; one is that dephosphorylation of VDAC by GSK-3 inhibition alters channel conductance directly, and the other is that GSK-3 inhibitors increase Bcl-2 binding to VDAC affecting the OMM transport. Besides, cardiac IR injury also enhances the activity of VDAC via promoting protein tyrosine nitration in VDAC (Yang et al., 2012).

Other new Ca^2+^ regulators located in ER–mitochondria microdomain have been reported. For example, GSK-3β could specifically interact with IP3Rs in ER–mitochondria microdomain, and subsequently increases the transfer of Ca^2+^ from ER to mitochondria, as well as sensitivity of cardiomyocytes to IR-caused apoptosis (Gomez et al., 2016). Additionally, mitochondrial chaperone cyclophilin D (CypD), a composition of mPTP, also cooperates with the VDAC1/Grp75/IP3R1 complex in cardiomyocyte (Paillard et al., 2013), enhancing ER Ca^2+^ efflux into mitochondria. The mitochondrial Ca^2+^ overload triggers excessive mPTP opening and thus initiates mitochondria-dependent cellular death in reperfusion-treated cardiomyocytes (Paillard et al., 2013). Conversely, a recent report suggested that mPTP opening modulates mitochondrial Ca^2+^ balance (Andrienko et al., 2016). This notion was initially confirmed by an earlier study that mPTP inhibitor, CsA, prevents mitochondrial Ca^2+^ efflux in adult rat ventricular cardiomyocytes (Andrienko et al., 2016), thereby postulating that mPTP may mediate mitochondrial calcium homeostasis. Altogether, the above information collectively suggest that moderate mitochondrial Ca^2+^ elevation governed by ER–mitochondria microdomain benefits cell energy metabolism and, however, uncontrolled mitochondrial Ca^2+^ accumulation, driven by ER–mitochondria microdomain in response to cardiac I/R injury, is detrimental to cardiomyocyte viability. Thus, preservation of mitochondrial Ca^2+^ balance via downregulating Ca^2+^-handling molecules in ER–mitochondria microdomain is an essential step to prevent the propagation of dangerous reperfusion signals.

Apart from mitochondrial calcium imbalance, cellular calcium overload also has the deleterious consequences on reperfused heart, which is highly handled with ER–mitochondria contact. The sarco-ER Ca^2+^ transport ATPase (SERCA), an ATP-driven protein, inversely transports Ca^2+^ back to the SR. However, in previous studies (Zhang Y. et al., 2016; Cui et al., 2018), the activity and expression of SERCA are statistically decreased in answer to cardiac I/R injury. The decreased SERCA is closely associated with cytoplasm calcium overload which obligates cardiomyocyte to mitochondria-dependent programmed death and finally amplifies reperfusion injury to heart either via triggering SR–Ca^2+^–XO–mitochondrial ROS axis (Zhu H. et al., 2018) or activating Ca^2+^–ROS–Drp1–mitochondrial fission pathways (Cui et al., 2018). Following study from Raturi et al. (2016) identified thioredoxin-related transmembrane protein 1 (TMX1) as a novel SERCA-inhibiting protein at ER–mitochondrial microdomains; inhibition of TMX1 may reduce the susceptibility of heart to I/R injury. Interestingly, the TMX1–SERCA complex formation could be enhanced by mitochondria-produced ROS (Krols et al., 2016). That is to say, mitochondria ROS may tighten up TMX1–SERCA interaction within ER–mitochondrial microdomains, effectively inhibiting SERCA activity. In traditional concept, mitochondria are the downstream effectors of ER via uptake of Ca^2+^ in ER–mitochondrial microdomains. However, their findings have established a new interactive mechanism in ER–mitochondria; damaged mitochondria could send a positive feedback to ER via ROS–TMX1–SERCA axis, further disrupting ER–calcium homeostasis and aggravating Ca^2+^ overload-mediated cell damage. However, no study is available to verify the feedback response between ER and mitochondria in cardiac I/R injury, and accordingly, further investigation is required to confirm this in acute cardiac damage model.

## Oxidative Stress

In response to reperfusion therapy, the restored blood rapidly re-introduces the fresh oxygen to the ischemic heart. Unfortunately, abundant oxygen would evoke a burst of reactive oxygen species (ROS) via multiple mechanisms reported by numerous studies (Zhou et al., 2015; Zhang Y. et al., 2016; Liu D. et al., 2017; Zong et al., 2017; Nuntaphum et al., 2018), leading to the cardiomyocyte oxidative stress. Cellular ROS is mainly produced by mitochondria when the electrons cannot be tightly coupled by the mitochondrial respiratory complex I and III (Hernansanz-Agustin et al., 2017; Miranda-Vizuete and Veal, 2017; Niaudet et al., 2017). However, other mechanisms have also been put forward to participate in this process, especially ER–mitochondrial microdomain. First, ER–mitochondrial microdomain could directly produce ROS via Ero1 (Gilady et al., 2010) and p66Shc (Lebiedzinska et al., 2009). Ero1, a key controller of oxidative folding and ER redox homeostasis, is enriched in ER–mitochondrial microdomain. Higher expression of Ero1 is closely associated with increased ROS production (Anelli et al., 2012). p66Shc (a 66-kDa isoform of the growth factor adapter Shc), a cytosolic adaptor protein related to ROS generation, could be detected in the ER–mitochondrial microdomain fraction (Patergnani et al., 2011). Careful examination from Lebiedzinska et al. (2009) revealed that the levels of p66Shc in the ER–mitochondrial microdomain is age-dependent and corresponds well to the mitochondrial ROS production. These data raise the possibility of a direct role for ER–mitochondrial microdomain in ROS outburst, which may be implicated in the cardiac I/R-mediated oxidative stress. Besides, in the repair stage of I/R injury or in the early phase of heart failure, the mitochondrial calcium overload mediated through the leaky RyRs increases the ROS production via NAD(P)H (Pacher et al., 2000). More importantly, the excessive superoxide in turn oxidizes the RyRs, thereby exacerbating mitochondrial calcium overload and ROS generation (Blackburn et al., 2017; Tomczyk et al., 2017). In consequence, this viscous cycle of Ca^2+^ leakage, mitochondrial calcium overload, and ROS outburst completely paralyzes cardiac contractility and obligates cardiomyocytes to apoptosis in the context of I/R injury (Gadicherla et al., 2017; Yang et al., 2017). Consistent with the above observations, following investigation further confirms that the ER-localized NADPH oxidase Yno1 is definitely required for cellular ROS accumulation in yeast (Leadsham et al., 2013). These pieces of information indicate that ER–mitochondrial microdomain-mediated ROS eruption is universal in many kinds of species.

More recently, in-depth study argue that ER–mitochondrial interface actually hosts a dynamic ROS nanodomain (Booth et al., 2016). At the molecular levels, ER–mitochondrial Ca^2+^ communication stimulates ROS mobilization from mitochondria to microdomain. It is the microdomain ROS transients rather than mitochondrial ROS overproduction sensitizes ER Ca^2+^ release to amplify Ca^2+^ oscillations (Booth et al., 2016). This piece of evidence fully updates our concept regarding microdomain ROS and verifies the existence of microdomain ROS for the first time. The difference between microdomain ROS- and mitochondrial ROS-triggered calcium imbalance is that the former requires lower concentration of ROS to oxidize ER–calcium channel. That is to say, the microdomain ROS may spatially and temporally confines or amplifies the mitochondrial superoxide anion production, which should be considered as the ROS switch and source. However, the detailed functional role of microdomain ROS is incompletely understood and little is known its function in the development and progression of cardiac I/R injury. Starting from these observations, further work is needed to explore the influence and mechanisms of microdomain ROS in cardiac I/R injury.

## Apoptosis and Necroptosis

The importance of cell death following IR injury is demonstrated in *in vivo* rodent model. Notably, prolonged periods of myocardial ischemia are related to an increase in the rate of apoptosis, whereas, paradoxically, reperfusion leads to an enhancement in necroptosis. There is more supportive evidence from our recent findings and other published data that most of cellular death could be blocked through inhibiting necrosis (or necroptosis), whereas only very marginal of reperfusion-induced cell death is attributable to apoptosis (Hochhauser et al., 2003; McCully et al., 2004; Yang et al., 2012; Zhang T. et al., 2016). Therefore, relieving cell death via preventing apoptosis and necrosis is vital to reduce I/R injury and improve the therapeutic efficiency of revascularization treatment. Many researchers have attempted to demonstrate the causal role of ER–mitochondrial microdomain in modifying I/R-mediated cell death. First, it is well documented that the sensitivity of cardiomyocyte to death (regardless of apoptosis and necroptosis) is fine-tuned by cellular calcium concentration (Mofid et al., 2017; Zhu et al., 2017; Pan et al., 2018) which drastically is affected by the ER–mitochondrial microdomain. Based on previous studies (Zhou et al., 2018d; Zhu H. et al., 2018), IP3R expression is upregulated in response to I/R stress, leading to the calcium overload in mitochondria. Subsequently, the calcium overload would activate necroptotic signaling in reperfused hearts via CaMKII–mPTP (Zhang T. et al., 2016) or XO–ROS–mPTP (Zhu P. et al., 2018) pathway. However, some other researchers argued that Ripk3-related cardiomyocyte necroptosis in I/R injury is not mediated through mPTP opening. They reported that suppression of autophagic flux contributes to cardiomyocyte death by activation of necroptotic pathways (Ogasawara et al., 2017). Actually, necroptosis is a kind of cell death program due to ATP depletion. Both mPTP opening and autophagic inhibition may interrupt the ATP supply, therefore exacerbating the reperfusion-mediated necroptosis. More recently, we provided partial evidence to confirm that ER-located IP3R is actually managed by Ripk3; genetic ablation of Ripk3 abrogates reperfusion-induced IP3R upregulation and ER stress (Zhu P. et al., 2018). These findings acknowledged the necessity of ER–mitochondrial microdomain in the excitation of Ripk3-induced necroptosis in cardiac I/R injury. However, we cannot exclude the protein interaction between Ripk3 and IP3R. If Ripk3 has the ability to directly integrate with IP3R, a new composition of ER–mitochondrial microdomain would be established, which means that the strategies to regulate the balance of Ripk3 and ER–mitochondrial microdomain could be a therapeutic target to cardiac I/R injury.

Besides, the downstream executive event of necroptosis is Ripk3-activated mPTP opening, which mediates the swelling and rupture of the organelle and cell due to the energy production disorder (Alghanem et al., 2017; Rossello et al., 2017; Rossello and Yellon, 2017). According to previous finding (Jahandiez et al., 2017; Zhou et al., 2017d), VDAC, one of the components of ER–mitochondrial microdomain, undergoes polymerization and resultantly promotes the hexokinase 2 liberation from mitochondria into cytoplasm. Hexokinase 2 is the endogenous inhibitor of mPTP opening and dissociation of hexokinase 2 from mitochondria has been shown to regulate, at least in part, cardiac I/R injury and mitochondrial integrity (Smeele et al., 2011; Pasdois et al., 2012; Nederlof et al., 2016).

Notably, other factors have also been reported to be involved in mitochondria-dependent cell death. Mitochondrial cardiolipin is a kind of phospholipid that predominantly embed in the inner mitochondrial membrane. The role of cardiolipin in the prevention of mitochondrial apoptosis and cardiac I/R injury is a well-established factor via repressing cyt-c liberation from mitochondria into cytoplasm (Brown et al., 2013; Shen et al., 2015; Ackermann et al., 2017). The cardiolipin downregulation and peroxidation would weaken the binding affinity of cyt-c to inner mitochondrial membrane and promote cyt-c leakage into cytoplasm (Zazueta et al., 2007; Zhang et al., 2010; Le Cras et al., 2017). Notably, although cardiolipin is synthesized by ER (Fleischer et al., 1967), the transfer of primarily phospholipids from the ER to mitochondria has been thought to be mediated via ER–mitochondrial microdomain (Gaigg et al., 1995). At the molecular levels, ER-mitochondrial encounter structure (ERMES) (Kornmann, 2013) is responsible for the cardiolipin exchange between mitochondria and ER. Structurally, ERMES is composed of mitochondrial distribution and morphology 10 (Mdm10), Mdm12, Mdm34, mitochondrial morphology 1 (Mmm1), and the regulatory subunit GTPase EF-hand protein of mitochondria (Gem1) (Klinkenberg et al., 2013). Functionally, ERMES possesses a synaptotagmin-like mitochondrial lipid-binding (SMP) domain that harbors an elongated hydrophobic groove in which different lipids can bind and possibly be transported (Kopec et al., 2010). Outside of cardiolipin transmission, ERMES also governs cardiolipin peroxidation via monitoring mitochondrial DNA (mtDNA) replication. Because the mitochondrial respiratory complex is encoded by mtDNA, the destruction of mtDNA inevitably suppresses the transcription and activity of mitochondrial respiratory complex. The decreased complex activity fails to capture free electron, finally evoking ROS outburst and subsequent cardiolipin peroxidation. In a word, the dysfunction of mtDNA copy is closely associated with cardiolipin oxidation. Interestingly, ER-resident protein Mmm1, one element of ERMES complex, structurally coimmunoprecipitates with Mgm101, a DNA-binding protein of the nucleoid, in chemically cross-linked mitochondrial extracts (Mbantenkhu et al., 2013; Pevala et al., 2016). This information proposes that a complex situated at the ER–mitochondrial microdomain has the ability to manage mitochondrial genome integrity and thus influence cardiolipin oxidation which facilitates the cyt-c liberation from mitochondria into cytoplasm under cardiac I/R injury. Interestingly, despite the established functional relationship between ER–mitochondrial microdomain and mtDNA over 10 years, little attempt is made to figure out whether microdomain-mediated mtDNA damage is one of the pathogenic factors for I/R injury.

## Concluding Remarks

**FIGURE 2:**
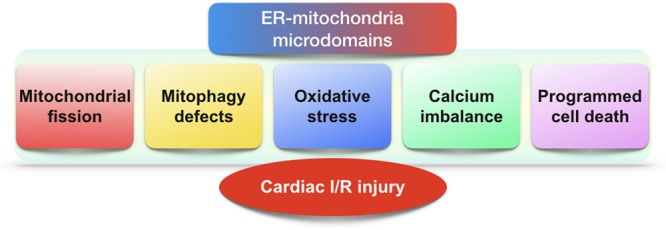
The role of ER–mitochondria microdomains in cardiac I/R injury. In the setting of cardiac I/R injury, excessive mitochondrial fission, defective mitophagy, oxidative stress, calcium dyshomeostasis, and programmed cell death are modulated by ER–mitochondria microdomains.

This review shows clearly that ER–mitochondria microdomain plays important roles in regulating cardiac I/R injury (**Figure 2**). The pathological interaction between ER and mitochondria promotes the malignant mitochondrial fission and inhibits the protective mitophagy. Thus, the ER regulates mitochondrial dynamics, and alterations in mitochondrial morphology uniquely reflect cell health. Interestingly, mitochondria are not only the downstream effector of microdomain; it also sends negative and/or positive feedback response to ER via microdomain. Accordingly, microdomain help ER and mitochondria shape the regulatory loop between them. Besides, ER and mitochondria also reciprocally transmit danger signals such as calcium overload and oxidative stress through microdomain which conveys organelle-extrinsic stress signals to promote cardiomyocyte death. Notably, little evidence is available for the precise role of ER–mitochondria microdomain in regulating I/R-initiated inflammation although NLRP3 infiammasome was found activated by ER–mitochondria microdomain (Zhou et al., 2011). Similarly, the relationship between ER–mitochondria microdomain and the cardioprotective signaling pathways including reperfusion injury salvage kinase (RISK) axis and survivor activating factor enhancement (SAFE) cascade has not adequately established, and therefore, more studies are required. Overall, in response to cardiac I/R injury, the ER–mitochondria microdomain represents a platform to modify the extracellular signal determining the degree of cellular insult. Based on this, therapies to influence the homeostasis of ER–mitochondria microdomain would be a therapeutic target to cardiac reperfusion stress in the clinical practice.

## Author Contributions

HZ, SW, SH, YC, and JR contributed to conception, drafted the manuscript, critically revised the manuscript, gave final approval, and agree to be accountable for all aspects of work ensuring integrity and accuracy.

## Conflict of Interest Statement

The authors declare that the research was conducted in the absence of any commercial or financial relationships that could be construed as a potential conflict of interest.
